# Cutting Pose Prediction from Point Clouds

**DOI:** 10.3390/s20061563

**Published:** 2020-03-11

**Authors:** Mark P. Philipsen, Thomas B. Moeslund

**Affiliations:** 1 Media Technology, Aalborg University, 9000 Aalborg, Denmark; tbm@create.aau.dk; 2 Danish Technological Institute, Gregersensvej 9, 2630 Taastrup, Denmark

**Keywords:** automation, pose prediction, point cloud, PointNet, meat production

## Abstract

The challenge of getting machines to understand and interact with natural objects is encountered in important areas such as medicine, agriculture, and, in our case, slaughterhouse automation. Recent breakthroughs have enabled the application of Deep Neural Networks (DNN) directly to point clouds, an efficient and natural representation of 3D objects. The potential of these methods has mostly been demonstrated for classification and segmentation tasks involving rigid man-made objects. We present a method, based on the successful PointNet architecture, for learning to regress correct tool placement from human demonstrations, using virtual reality. Our method is applied to a challenging slaughterhouse cutting task, which requires an understanding of the local geometry including the shape, size, and orientation. We propose an intermediate five-Degree of Freedom (DoF) cutting plane representation, a point and a normal vector, which eases the demonstration and learning process. A live experiment is conducted in order to unveil issues and begin to understand the required accuracy. Eleven cuts are rated by an expert, with 8/11 being rated as acceptable. The error on the test set is subsequently reduced through the addition of more training data and improvements to the DNN. The result is a reduction in the average translation from 1.5 cm to 0.8 cm and the orientation error from 4.59° to 4.48°. The method’s generalization capacity is assessed on a similar task from the slaughterhouse and on the very different public LINEMOD dataset for object pose estimation across view points. In both cases, the method shows promising results. Code, datasets, and other materials are available in Supplementary Materials.

## 1. Introduction

The replacement of human muscles and minds across all sectors with repetitive, dangerous, and dirty jobs attracts much interest from both governments and industry. Such jobs are often found in the construction and food industry. Unfortunately, some of the most demanding and dangerous jobs are also proving to be the most challenging to automate. The difficulty of automating these jobs is explained by Moravec’s paradox. It observes that machines with relative ease can be made to perform tasks that we perceive as complex or difficult, while great difficulty is associated with automating tasks that, on the surface, seem intuitive and easy [1]. This might stem from the fact that perception and motor skills seem easy because they have been acquired through more than a billion years of evolution, while more abstract skills like math and logic only recently have become relevant. The success in automating tasks involving math, logic, and memory lends itself to the fact that such tasks more readily break down into steps that can be put into a program. Unfortunately, the same is not the case for perception and motor skills. Machine Learning (ML) and most noticeably Deep Learning (DL) pose a solution that circumvents the problem of having to specify concise algorithms. Instead, programs are learned from experience. However, the resulting programs may exhibit problems with generalizing to unseen examples. This is manifested by a sensitivity to what should be inconsequential changes in the input [2] and difficulties in recognizing otherwise known objects when encountered from a new point of view [3]. These weaknesses can, to a large extent, be overcome by supplying sufficient examples during the training phase. The required number of examples is highly dependent on the type of task, and their acquisition is likely to be a major hurdle. For this reason, most successes in ML have so far been demonstrated under controlled conditions and with tasks where vast amounts of data are readily available. When solving complex problems, such as learning to interact with natural objects, the range of variation and thus the required amount of examples quickly becomes difficult to anticipate and obtain. Data acquisition might require domain experts for labeling, and the labeling task itself might be difficult and ambiguous. Furthermore, the valuable labels and examples may become outdated with time. This makes application areas such as medical imaging notoriously data starved [4]. The same problems are also characteristic in the development of new slaughterhouse automation where the source of variation is near endless, labeling is challenging and expensive, processes are destructive and non-reversible, and products change over time.

Slaughterhouses are surprisingly diverse in terms of what machines are used, what is automated, and how the production is laid out. The machines are highly specialized and typically rely on simple measurements combined with extensive mechanical fixtures. The production typically follows a highly optimized line production paradigm, where optimization efforts have been focused on increasing throughput by pushing employees and machines to their limits. This comes with the unfortunate side-effects of increased vulnerability to breakdowns, lack of flexibility, compromises to yield and quality, and a high frequency of injuries among employees because of the frequent repetition of a limited set of movements. This poses a challenge to machine producers and slaughterhouses when attempting to introduce new automation. The result is too often that machines have to be customized to the individual slaughterhouse or that new solutions are turned down over concerns with the integration into the existing production. A transition from line production to cell-based production has the potential to alleviate many of these concerns by replacing specialized machines with industrial robots and introducing a much larger degree of parallelism and redundancy. Efforts in this direction are currently underway through research projects such as the Norwegian “Meat 2.0” project [5] and the Danish “Augmented Cellular Meat Production” project [6].

The work presented here is part of achieving the overall goal of building robot controllers for the new robot cells. These controllers cannot rely on the same degree of mechanical fixation and must cope with significant variation by relying on advanced sensors and ML. The specific goal of the work presented in this paper is to exploit recent advances in the application of deep learning for obtaining an understanding of 3D objects.

### 1.1. Case

**Figure 1:**
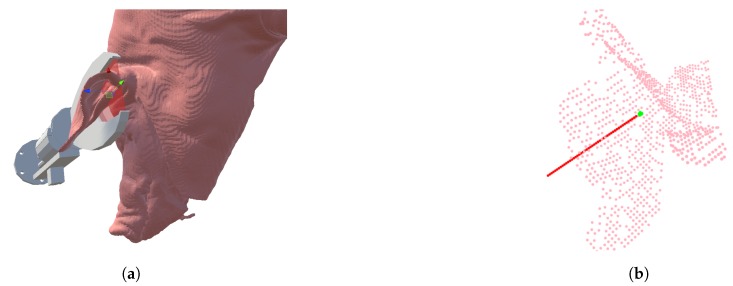
(**a**) Correct tool pose illustrated using 3D models of the tool and pig. (**b**) A point cloud of the ear region with our representation of a 5-DoF cutting plane consisting of $P_{0}$ (green) and $\vec{nz}$ (red).

The specific task, selected as the basis for this work, is the simple yet challenging slaughterhouse operation of removing pig’s ears using hydraulic scissors. The scissor used here is similar to the ones used by slaughterhouse workers. It is shown in Figure 1a, modeled in Virtual Reality (VR), and in Figure 15c, mounted on a FANUC M-710iC robot. Ear cutting is still performed manually, and involves placing the large scissors accurately around the ears. The task only involves a single cutting pose, but the variation in pig ears, as well as the high accuracy requirements make it difficult to automate. The variation comes from the variability in presentation and variations in shape and size. The presentation of pigs can be controlled to some degree, but variation in position and orientation is inevitable. The natural variations, most noticeably, include the posture of the ear, which can appear erect, flopped, or an intermediate of the two [7].

A correct placement of the scissors is illustrated in Figure 1a. The cut must be placed along the translucent red plane between the scissors’ blades. The cutting pose is largely agnostic to the yaw, i.e., rotation around the tool’s z-axis. This makes consistent demonstration of six-Degree of Freedom (DoF) poses both difficult and unnecessary. It is thus desirable to retain the freedom to determine the tool’s orientation around its own z-axis at a later stage. For this reason, an intermediate 5-DoF cutting pose representation is proposed, namely a point $P_{0}$ and a normal vector $\vec{nz}$, corresponding to the direction of the tool’s z-axis. This representation is illustrated in Figure 1b where $P_{0}$ is shown as a green dot and $\vec{nz}$ as a red line on top of a point cloud from the ear region.

**Figure 2:**
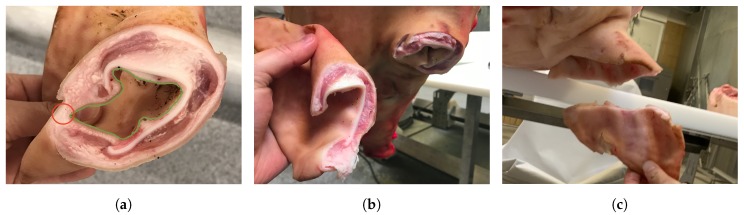
(**a**) Borderline, too close to the head. (**b**) Good. (**c**) Bad, too far from the head.

An optimal cut is highly dependent on the anatomy of the individual ear. The cut must be roughly parallel to the head and far enough away such that the ear canal is separated. A borderline acceptable result is shown in Figure 2a. Here, the ear canal, outlined in green, is still connected by skin. Ideally, the area marked by a red circle would be left on the head. Figure 2b shows a good cut where this is the case, and Figure 2c shows a terrible cut, where much the valuable ear is left on the head.

### 1.2. Related Work

Pose prediction for robotic manipulation typically begins with localizing objects of interest. This is done using either template matching [8,9], bounding box detectors [10], or segmentation methods [11,12,13]. Segmentation methods seem to be the superior choice because of the more accurate and richer basis for extracting information. This is especially the case in cluttered scenes where bounding boxes prove too crude [13]. Template-based methods are notoriously slow and may cover the variation insufficiently. After localization comes extraction of object representations, either an intermediate description of the objects or simply the pose of the object. With the template-based method, the matching template reveals the pose either through the transformation used to generate the template or the transformation needed to fit it. Examples include rendered images of objects with known poses that are matched to observations using a CNN [14] and 3D scans of known objects fitted to segmented objects using an Iterative Closest Point (ICP) algorithm [12,15]. Key points are a popular intermediate representation that offers better flexibility in terms of handling variation. Their downsides include challenges in coping with occlusion and potential difficulty in labeling. A recent approach predicted the 2D image plane locations of the vertices from the object’s 3D bounding box [16]. A similar approach that is better suited for cluttered scenes divides images into patches where the corresponding object and 2D projections are predicted for each patch. Predictions are propagated across patches and used to build a robust set of 3D-to-2D correspondences [17]. Finally, feature representations are widely used. Historically, features have been handcrafted to describe image features such as edges, textures, and colors, as seen in [18]. For depth images, Height Accumulated Features (HAF) are an example of handcrafted features derived from the average heights in small regions. They are useful for describing the object’s shape, as well as the surrounding space [19]. More recently, feature-based methods have begun relying on DNNs. An early example employs a two-step cascaded approach, where a small DNN finds grasp candidates and a larger DNN selects the best candidates [20]. Examples also include the reuse of convolutional layers from an ImageNet-trained classification networks, where only the fully connected layers are trained for grasp prediction [21]. Another method uses two ResNet-50 networks in parallel for extracting features from RGB images and depth images. Features extracted from the ResNet-50 networks are merged before being passed into another final CNN that predicts grasp configurations [22]. While the feature-based methods typically output the wanted pose directly, methods that produce intermediate representations must employ a final step to produce usable poses. For going from key points to 6-DoF poses, this is typically achieved using a Perspective-n-Point (PnP) algorithm [16,17]. A recent comprehensive survey that covered the different aspects involved in robotic grasping can be found in [23].

Having briefly covered the main directions and techniques for pose prediction in general, we take a look at systems that specifically address natural and deformable objects. The application areas include agriculture, household automation, food processing, and human-machine interaction. In these cases, many of the techniques used for rigid man-made objects fail due to the added variation of deformable and natural objects [24]. The first example addresses the problem of detecting and estimating the pose of fruits. A Fully Convolutional Network (FCN) is used to segment fruits and branches in RGB images. The output of the segmentation is clustered to detect the individual fruits, and a 3D line segment detection method is used to reconstruct branches. The 3D pose of the fruit is then estimated using the fruits’ center and branch information [25]. Recent methods trend towards end-to-end approaches where tool poses are predicted directly from sensor data without explicit intermediate representations. These methods essentially treat pose prediction analogously to object detection. An early attempt in this direction learns object agnostic grasp prediction for household objects. Pose hypothesis are generated based on heuristic sampling strategies presented in an earlier work [26]. Candidate selection is performed by a CNN based on representations of the local surface geometry. In order to get the point cloud information into a suitable representation for a CNN, the voxels are projected onto planes orthogonal to the basis axis of a voxel grid [27]. A completely end-to-end approach is used for teaching a robot to grasp real fish. A 3D CNN predicts grasps from a 3D occupancy grid of a box filled with fish. The occupancy grid can be generated from multiple viewpoints, depending on the number of depth sensors available. Training is done exclusively from synthetic data and a low number of demonstrations made in VR, which provides an intuitive interface for demonstrating grasps. Compared to their previous work [28], domain randomization is added, and the learned model is transferred to a real robot. The limitations of the used voxel grid representation is evident and leads to a difficult balance between the size of the receptive field, resolution, and the number of parameters [29]. Fortunately, these problems are mostly solved with the introduction of PointNet [30], which makes the application of DL to unordered sets of points possible. Until very recently, pose prediction methods required the point cloud to be projected onto multiple 2D images [27] or rasterized into dense 3D volumes [29]. PointNet achieves this by transforming individual points to a high-dimensional space and applying max pooling. The result is a permutation invariant feature vector that describes the point cloud. The PointNet architecture forms the basis for much recent work; this includes methods for evaluating grasps from raw point clouds, as seen in [31]. Here, grasp candidates were sampled based on heuristics before being evaluated using a PointNet-style network. PointNet has also been used to generate per-pixel embeddings as a step in a large pose prediction pipeline [13]. The problem of regressing hand key points is being tackled by an extension of the PointNet architecture that addresses some of PointNet’s limitations by creating a hierarchy of features [32].

### 1.3. Contribution

The purpose of this work is to apply recent advances in pose prediction to an industrial automation problem that differs in important ways from the classical pose prediction tasks. We evaluate how well a DNN, based on the PointNet architecture, can learn to predict tool poses from human demonstrations. We propose an intermediate 5-DoF pose representation that simplifies the demonstration and learning process for our task. A live test of the system is performed in order to uncover issues and give an idea of the required accuracy. The DNN’s ability to make accurate predictions is compared to the accuracy of human demonstrations. Finally, possible directions for improvement are suggested. The contributions of this work are thus:

1. A framework for pose prediction from point clouds.
2. A novel 5-DoF pose representation for a specific category of tasks.
3. Considerations and future work for pose prediction in a challenging setting.

## 2. Materials and Methods

**Figure 3:**
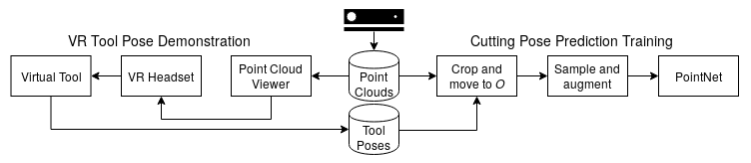
Training system components.

**Figure 4:**
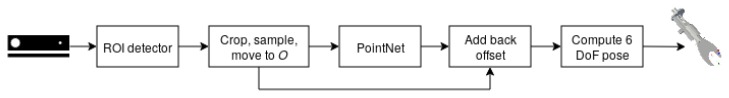
Inference system components.

Our pose prediction framework can be considered as two separate systems: a training system for collecting point cloud and tool pose pairs as well as learning a mapping from the point cloud to tool pose; another system for performing inference based on the learned mapping and sending actionable 6-DoF poses to a robot equipped with the cutting tool. Both systems share several of the same components, but also include components that are only relevant to the given system’s task. In this section, we describe details of the components that make up the two systems. The components involved in the training system and the inference system are shown in Figure 3 and Figure 4, respectively.

**Figure 5:**
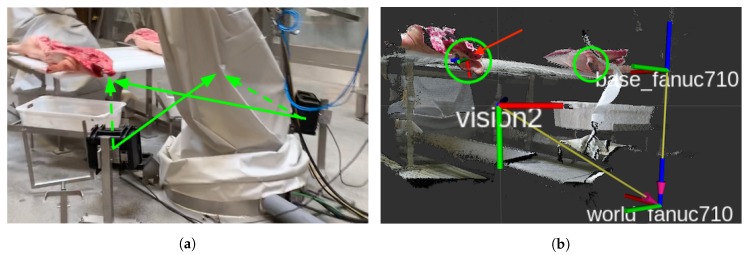
(**a**) Dual camera setup, one on each side of the robot. Highlighting the line of sight to ears from each camera. (**b**) Point of view of the left camera. Camera frame’s relative position to the world frame. Visible ears marked by green circles. Risk of occlusion from snout marked by a red arrow.

The point clouds, used by both systems, were captured using the setup shown in Figure 5. Point cloud and tool pose pairs were created by visualizing RGB point clouds from a Kinect V2 in a HTC VIVE VR headset. Here, the user could see and move around the point clouds in order to get a feel for the geometry of each ear. Using virtual scissors, the desired cutting poses were demonstrated by placing the scissors on top of the point cloud visualization. Based on the demonstrations, points around the ears were cropped from the full point cloud. The crops were then centered around the origin *O* of the coordinate system. This transformation could easily be reversed and eliminated the need for learning invariance to the overall placement of points in the camera space. A fixed number of points was needed as the input, and since the number of points in a crop depended on the individual ear, the view point, and the distance, the number of points varied greatly. A fixed number of points were selected based on random sampling, producing point clouds with the same cardinality. The sampled point clouds along with the demonstrated cutting planes were used to train a DNN to regress cutting planes from point clouds.

The inference system could not rely on annotations for determining a Region Of Interest (ROI) and thus needed an ROI detector. An off-the-shelf bounding box detector served this purpose. At inference time, the translation offset used to center the point cloud crop around *O* was added back, such that the position prediction was placed correctly in the world. Finally, the 6th undetermined DoF was computed in relation to the robot. In the following, we describe theses subsystems in more detail.

### 2.1. Sensors

The system relied on point clouds captured by a Kinect V2. With its time-of-flight technology and advanced ambient light compensation, it has long been the superior choice in terms of resolution, robustness, and accuracy. This is however quickly changing with its discontinuation and the wealth of new sensors becoming available. One promising candidate for its replacement is its successor, the Azure Kinect, which improves on Kinect V2 in most regards. For this work, point clouds were captured in their native resolution of 512 × 424 using the IAI Kinect2 ROS package [33]. The camera setup is shown in Figure 5a,b. Two cameras were mounted on the floor, one on each side of the robot. Both ears, marked by circles in Figure 5b, were mostly visible in either camera; however, the placement and softness of the pigs varied, and there was a risk of self-occlusion of vital parts. Specifically, the base area of the ear might be occluded. This was somewhat the case with the nearest ear in Figure 5b, where the snout, marked by the arrow, covers some of the ear.

**Figure 6:**
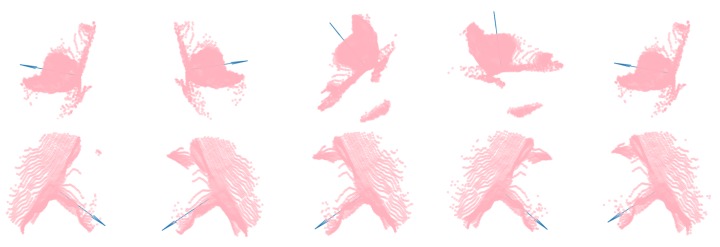
Point cloud crops shown with arrows representing the demonstrated cutting planes.

From experience, visual information covering both sides of the ear is helpful for accurately determining where to place the cut. However, in this work, only a single point of view was used. The other camera was exclusively used to gather additional data and to keep the option for combining point clouds from multiple views open at a later stage. Figure 6 shows a selection of point cloud crops with arrows illustrating demonstrated cutting places. The top row consists of examples from the nearest ears, and the bottom row shows examples from the ears furthest away from the cameras. It was clear that the ears looked vastly different based on the view point. Additionally, they contained noisy depth measurements and artifacts along edges, and the points were unevenly distributed across the area of interest.

Data were collected in two phases. The first samples were collected before a fixed setup was ready by a single camera mounted on a movable tripod. This meant that these samples differed somewhat from the rest and contained some variation in the viewpoint. This dataset was used for validation. The remaining data were collected using the fixed dual camera setup and were split between the training and test set. According to convention, the slightly abnormal data from the initial data collection would have been divided among all three datasets. However, we chose not to do this since the camera setup was likely to undergo future adjustments and we wanted to select the model that best generalized to such changes using a more varied validation set. We also wanted to know the performance of the model for the current setup, and for this reason, the test set was limited to the most recent samples. Table 1 shows an overview of the content of each of the three datasets.

The training and test sets contained data from both cameras. For this reason, there were around twice the number of ears counted as from a unique view point, compared to the number of unique ears. The validation set consisted of data collected from a single camera, and therefore, the number of unique viewpoints was only slightly higher than the number of unique instances. The number of frames denoted the frames that were captured. Some frames might contain less than two visible ears, and some pigs were captured twice from the same point of view. This was also the reason that the number of samples was higher than the number from a unique viewpoint.

### 2.2. Tool Pose Representation

A tool pose is generally defined by 6-DoF specifying the position and orientation of the tool in a 3D space. The tool may add additional DoFs depending on its configurability [34]. For some problems, one or more DoF can be kept fixed as seen with some widely used tool pose representations. A popular representation is defined by g=f(x,y,h,w,θ), position x,y, height *h*, width *w*, and orientation with respect to the horizontal axis of the image plane θ [22,35]. Here, the two additional variables needed to specify a complete tool orientation in 3D were kept fixed. Further reduced examples, such as x,y,w,θ [20], x,y,z,θ [36], and x,y,θ [21], were also used. The popularity of these representations comes from their suitability for methods that operate on images. Recently, pose prediction methods have begun to break free from the restrictions of the image plane and instead represent pose as position and either Euler angles [37], quaternions [37,38], or normal vectors [29]. Each of these representations of rotation is associated with properties that can impede a learning algorithm. Euler angles wrap from 2π radians to 0 and suffer from gimbal lock; quaternions must satisfy the unit-length constraint; and normal vectors suffer from the orthonormal constraint of the rotation matrix. A representation based on Lie algebra promises to solve these issues by representing rotations in a continuous space parameterized by the three scalars in a skew-symmetric matrix [39].

For the purpose of representing a tool pose in the ear cutting application, it is preferable to represent poses with only 5-DoF. As explained in Section 1.1, the tool pose needed to perform a cut is largely agnostic of the orientation around the tool’s z-axis. This makes consistent labeling difficult, and the inconsistency is likely to impede the training process. For this reason, the 6th DoF was disregarded and instead computed analytically after the DNN made a prediction. This is described in Section 2.7. Since the cutting plane could be represented by a point $P_{0}$ and a single normal vector $\vec{nz}$, a simple and effective solution was to use a reduced version of the representation used by [29]. The reduced 5-DoF version also did not suffer from the orthonormal constraint that applied to the full 6-DoF representation, which consisted of multiple normal vectors. This 5-DoF “point-normal” representation is applicable to applications where the orientation around one of the tool’s axis is not crucial or can be trivially determined by other means. This is typically the case when the tool is isotropic such as many pick-n-place tasks where suction cups are used.

(1) $$CuttingPlane=\{P_{0},\vec{nz}\}$$

### 2.3. Demonstration in Virtual Reality

**Figure 7:**
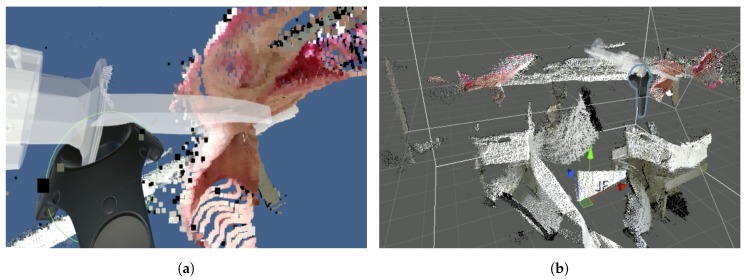
Cutting pose demonstration in VR. (**a**) View in the VR headset. (**b**) Scene overview.

The programming by demonstration paradigm used with collaborative robots has lowered the difficulty of robot programming significantly. The same paradigm can be used to train robots by Learning from Demonstration (LfD). By utilizing mixed reality environments, these approaches become available for all types of robots. Using a HTC VIVE VR headset, point clouds were visualized, and an expert demonstrated the cutting poses. ROS# [40] was used as a bridge for transmitting point clouds to the Unity game engine [41], which interfaced with the VR headset. The VIVE controllers let the expert demonstrate cutting poses by grabbing a 3D model of the cutting tool. The tool was then placed at the appropriate location using the point cloud visualization as guidance. This process is shown in Figure 7a,b. The controller’s buttons allowed for saving cutting poses and switching between point clouds. VR is an intuitive interface for demonstrating tool poses [29], and its use is further justified and described in our prior work [42].

### 2.4. Region of Interest

**Figure 8:**
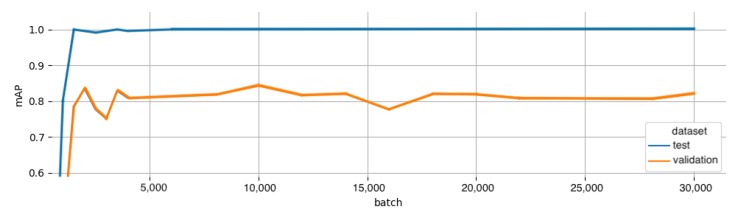
(Orange) mAP for the test set. (Blue) mAP for the validation set.

The optimal ROI detector for our task would predict object centers only and not be punished for a poorly sized bounding box. However, this consideration was not critical, and an off-the-shelf bounding box detector was used for detecting ears instead. The requirement for the ROI detector was that it quickly and robustly provided image coordinates for the centers of any ears in the scene. The YOLO [43] detector served this purpose, specifically the latest and fastest incarnation, tiny YOLOV3 [44]. YOLO is an end-to-end CNN, which divides the input image into a fixed grid. Bounding boxes, labels, and confidence scores are predicted for any objects appearing inside each cell. Figure 8 shows that the detector converged to very good performance after only a few thousand batches of training examples. The evaluation metric was the mean Average Precision (mAP), which is widely used for evaluating bounding box detectors. Training was done using pre-trained weights from the COCO object detection dataset [45] and with a batch size of 64. Based on the performance on the validation set, the weights at batch 10,000 are selected. Performance is significantly worse for the validation set than for the test set. This supports our assumption that the validation set is more challenging and a bit of an outlier.

### 2.5. Point Cloud Preprocessing

To prepare the input for a PointNet-style neural network, the following steps were found to be necessary or beneficial. (1) The input should contain primarily relevant points. (2) The input point cloud must contain a fixed number of points. (3) The input should look similar independent of the distance between the object of interest and the sensor.

#### Cropping

Using either the 3D coordinate for the center pixel of the detected bounding box or the demonstrated tool position, points inside a radius *r* were cropped from the full point clouds. While ears did vary noticeably in size, a sphere with *r* = 0.15 m empirically proved to contain points from a satisfactory volume that ensured that the relevant points were captured and most unwanted points were filtered out. Figure 6 shows a few examples of point cloud crops containing points from both head and ear.

#### Sampling

**Figure 9:**
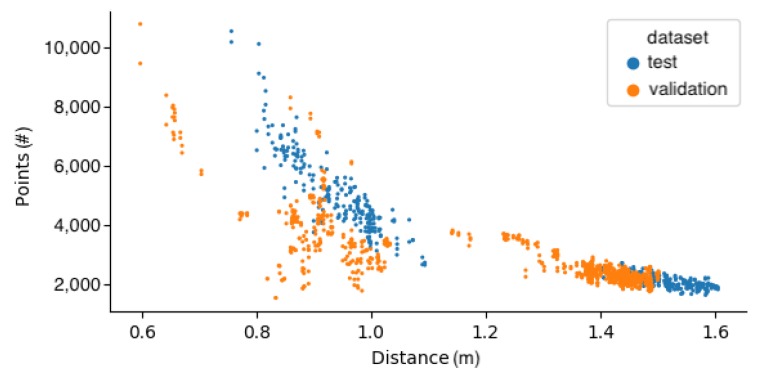
Number of points in crops relative to the distance from the camera.

A strict requirement for using PointNet-style [30] neural networks is a fixed size input. This is usually achieved by sampling the required number of points from meshes, using the farthest point sampling. If a mesh is available, it enables the sampling of point clouds with arbitrary sizes [30]. Another example is random sampling, as used in [46], where 1024 points were sampled from 1200 points during both training and testing; this was done partly as an augmentation technique. Typically, the number of points lies in the range of 512–4096. In our case, a fixed number of points was acquired from the point cloud crops through random sampling. This simultaneously ensured that the point clouds looked similar independent of the range from which they were captured. To determine an appropriate number of points to sample, we looked at the minimum number of points in the point cloud crops. In Figure 9, the number of points in the crops is shown in relation to their distance from the camera. Examples from the training set were left out because they followed a similar distribution as the test set. Again, it was clear that the validation set was noticeably different and more varied.

**Figure 10:**
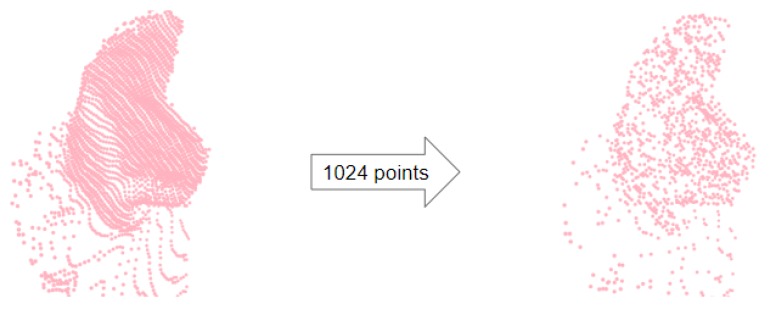
(**Left**) Point cloud cropped from the sphere of interest. (**Right**) Random sampling of 1024 points.

The sample size should be safely below the minimum number of points that can be encountered, as well as high enough that sufficient coverage of the region can be ensured. All of the crops contained significantly more than 1024 points, which seemed like a reasonable number. The result of sampling 1024 points from a crop is shown in Figure 10.

#### Augmentation

The first type of augmentation to be employed was mirroring across the x-axis, effectively doubling the amount of samples. The next was random perturbation of center points, resulting in small offsets in the point cloud crops. This also served to compensate for inconsistencies in the localization of ears. Finally, random sampling was considered as data augmentation [46], a nice side-effect of the primary purpose of selecting a fixed number of points. Augmentation by rotation, scaling, and adding noise did not result in improvements and was not used.

### 2.6. Cutting Pose Prediction

There is a recent, yet clear, consensus that point clouds are the data representation of choice for vision systems that deal with 3D data. Point clouds are the canonical representation of 3D objects. They contain the maximal amount of information that can be extracted from the object through LiDAR or depth sensors. PointNet [30] is the seminal work in applying deep learning to point clouds. The primary challenge is achieving invariance to permutations. This can be done by using symmetric functions, where the result is the same irrespective of the order of the input. With PointNet, this is shown to work if the individual input points are mapped to a high-dimensional space before channel-wise max pooling is used to achieve permutation invariance by aggregating the point features into a global feature vector. Because PointNet is intended for classification and segmentations tasks, it includes Spatial Transformer Networks (STN), which learn to transform either input points or intermediate features such that invariance to geometric transformations, e.g., rotation, is learned.

**Figure 11:**
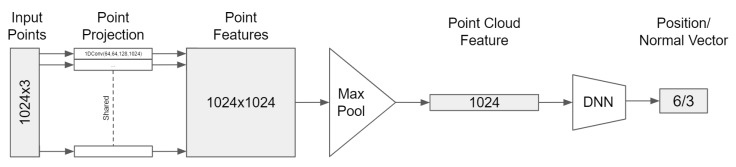
Cutting pose regression network based on PointNet.

PointNet was the basis for our architecture for regressing cutting poses from point clouds. We left out the STN module since invariance to geometric transformations was unwanted for pose prediction. The softmax activation functions were replaced by linear activations that output three values for either position $P_{0}$ or normal vector $\vec{nz}$. The modified architecture is shown in Figure 11.

The loss function used for training the network was the Mean Squared Error (MSE). It weighs large discrepancies relatively severely, while small discrepancies much less. This is desired with supervised learning from human demonstrations of tool poses, since the demonstrations are likely to contain small inconsistencies, but are never completely wrong.

### 2.7. Cutting Pose → 6-DoF Tool Pose

The prediction of $P_{0}$ and $\vec{nz}$ defined five out of six needed DoF. To perform the cutting operation, a full 6-DoF pose was necessary. The final DoF was determined by the addition of a normal vector $\vec{nx}$, based on the the cross-product of $\vec{nz}$ and a static vector $\vec{rob}$. $\vec{rob}$ was determined by the setup of the robot cell. The last normal vector $\vec{ny}$, needed to define a 3D rotation matrix *R*, was found from the cross-product between $\vec{nz}$ and $\vec{nx}$. The rotation matrices were converted to a quaternion representation *Q*. This could be done using a number of methods [47]. The Sarabandi–Thomas’ method [48] provides improved numerical stability compared to the widely used Shepperd’s method through an increase in the number of alternative solutions. The result was a 6-DoF pose $\left\{P_{0},Q\right\}$.

## 3. Results

The following experiments served to demonstrate the system’s performance, guide future applications of this or similar methods, and explore potential improvements. Below is a list of experiments addressing each of these aspects of the contributions. It is followed by an overview of the different metrics that were used in related work for evaluating pose prediction methods. Before delving into the individual experiments, the metric used in this work is thoroughly introduced.

- Cutting pose prediction performance.
- Network architecture design choices.
- Impact of training set size.
- Live test in the robot cell.
- Demonstration quality.
- Future applications.

The performance of pose prediction methods is typically evaluated using one of the following measures of the error between predictions and the ground truth: The Average Distance (AD) metric measures the distance between vertices of a 3D model with the estimated pose and one with the ground truth pose [8]. The ADD metric computes a score based on AD and the relative size of the object [16]. Similar to AD based on 3D vertices, model vertices can be projected into the image plane where AD is measured in pixels. This is particularly useful for applications such as augmented reality, where it is the pose representation in a 2D image that is important. An even simpler measure is the Intersection over Union (IoU) criterion, which is widely used for evaluating object detectors. It measures the degree of overlap between the bounding boxes around objects derived from their predicted pose and the ground truth [49]. Finally, translation and rotation error are widely used. For an industrial assembly task, accuracies of <1 mm and <1° were reported [50]; the prediction error in the pose of automotive objects was 1 cm and <5° [12]; pose estimation for industrial work-pieces achieved an accuracy <10 mm and <2° [51]; and for a bin-picking task, translation error was <23 mm and rotation <2.26° [52].

These measures, by themselves, do not necessarily say much about the performance for actual tasks. The required accuracy is highly dependent on the case and other factors such as tool type and object characteristics. In many cases, the accuracy measures are accompanied by a success rate based on thresholds determined through experimentation or derived from experience. A pose might be considered correct if AD is below 10% of the object diameter [8], if the AD of the projected 2D key points is <5 pixels [49], or if the IoU of projected bounding boxes is above 0.5 [49]. A translation error below 5 cm and rotation error lower than 5° are deemed acceptable for predicting the pose of a camera in a known 3D scene [53]. Successful grasps can also be modeled in a probabilistic framework by sampling the pose error space and determining outcomes [54].

While MSE is ideal as training loss, it is not great at conveying performance in a direct and interpretable way. The Mean Average Error (MAE) is a more understandable measure of error and is used for comparing results during experimentation. MAE is superior in conveying error, but produces gradients with constant magnitudes, which is a drawback when learning using back-propagation. For position predictions, MAE is the average distance in meters between the predicted $P_{0}$ and the demonstrated $P_{0}$. For normal vector predictions, MAE is the angle between the predicted $\vec{nz}$ and the demonstrated $\vec{nz}$.

### 3.1. Cutting Pose Prediction Performance

When evaluating model performance on the test set, the MAE was 0.008 m for $P_{0}$ with a standard deviation of 0.004 m and 4.481° for $\vec{nz}$ with a standard deviation of 2.692°. Table 2 shows the mean error for each of the three datasets.

**Figure 12:**
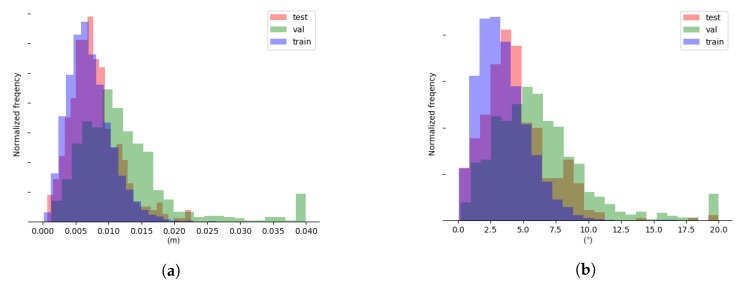
(**a**) Distribution of position errors. (**b**) Distribution of normal vector errors.

In addition to quantifying prediction accuracy, a comparison of performance across training, validation, and test sets may provide insights into the potential for improvement and issues with the datasets. The distributions of prediction errors that formed the basis for the results presented in Table 2 are shown in Figure 12. It should be noted that a few outliers were collected in bin 0.04 in Figure 12a and in bin 20.0 in Figure 12b, respectively. This was done for presentation purposes.

**Figure 13:**
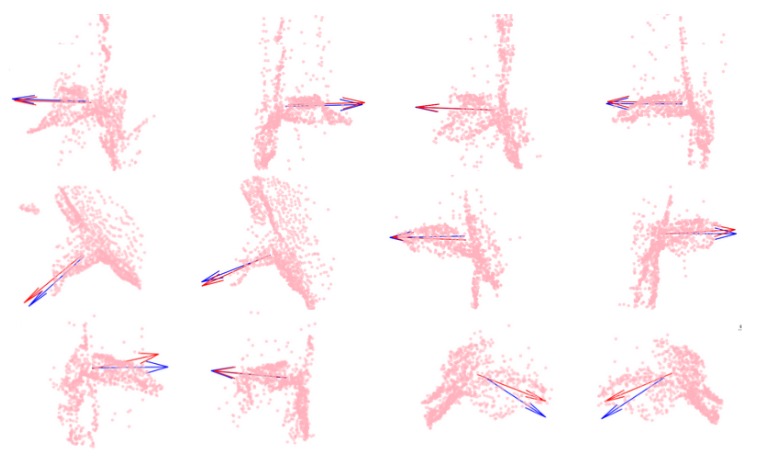
Test samples with arrows representing the demonstrated (blue) and predicted (red) cutting planes. (Top row) Samples with the smallest prediction error for $\vec{nz}$. (Middle row) Samples with median prediction error for $\vec{nz}$. (Bottom row) Samples with maximal prediction error for $\vec{nz}$.

In order to visualize the severity of a given error in predicting $\vec{nz}$, the four best predictions are shown (top row) in Figure 13 along with four examples around the median (middle row) and the four worst predictions (bottom row).

### 3.2. Network Architecture Design

There is a multitude of options when designing the DNN. Here, we focused on the degree of separation between the prediction of the three components of the position $P_{0}$ and the three components of the normal vector $\vec{nz}$. One option was to produce a unified 6D output where the first three variables belonged to $P_{0}$ and the last three belonged to $\vec{nz}$. Another option was to split the DNN at some point before the output, thereby producing two separate outputs, each consisting of three variables. This option comes in a number of variations depending on where the split was made. Typically, DNNs for pose predictions diverge into separate branches for position and orientation [38,55]. To balance the loss for position and orientation errors, a scale factor β can be used [55]. The tricky balancing of multiple losses can be avoided by using completely separate predictors for position and orientation [11].

Here, three architecture variations were tested. The variants differed based on the place where the network separated the connections to the outputs. For the “Full” split network, two separate and identical networks of the type seen in Figure 11 were combined at the output. The “Early: split network separated after a shared point cloud feature by splitting into two DNNs, one for $P_{0}$ and one for $\vec{nz}$. Finally, with the “Late” split network, a shared DNN was processing the shared point cloud feature before finally splitting into two separate DNNs. Table 3 shows the validation set performance for the different variations of the DNN architecture. The best performing architecture, “Full”, was used for producing results throughout the paper.

### 3.3. Training Set Size Impact

**Figure 14:**
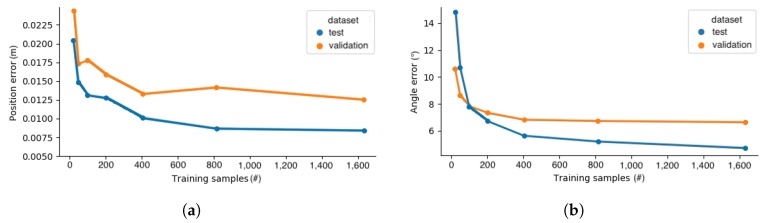
Number of training samples vs. (**a**) MAE position error. (**b**) MAE normal vector error.

Since data collection is expensive and time consuming, it is interesting to attempt to determine whether worthwhile improvements are attainable by expanding the training set. By withholding fractions of the training set and training models using increasing fractions of data, it is possible to gauge expected gains from, e.g., a further doubling of the training set. It has previously been found that there was a logarithmic relationship between the performance and the amount of training data in computer vision tasks [55]. Figure 14 shows the improving, but diminishing returns with increasing training set size.

### 3.4. Live Test

**Figure 15:**
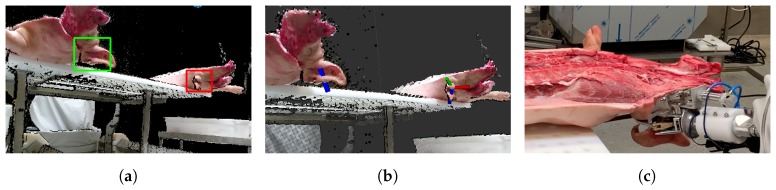
Test highlights. (**a**) Localization of ears. (**b**) Pose prediction. (**c**) During execution of the cut.

Before the complete training and test sets were collected, a live test was conducted to investigate the requirements and needed accuracy for performing acceptable cuts. This meant that the predictions were based on a preliminary model. Some highlights from the test are shown in Figure 15.

**Figure 16:**
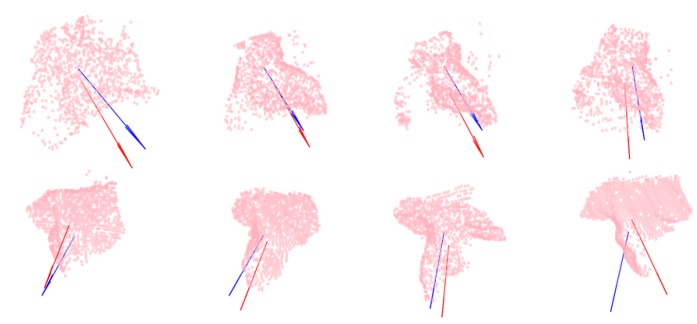
Predictions (red) on data from the live test shown along side human demonstrations (blue).

Both left and right ears were cut based on the prediction made from the point of view shown in Figure 15a,b. The result of each cut was rated by a butcher on a scale of 1–5. This was the same rating scale previously used and described in [42]. A score of 1 was given to unacceptable results, 3 as acceptable, and 5 as perfect. A total of 11 ears were cut and rated. The result was that 8/11 cuts were rated as acceptable or better. In order to illustrate the severity of a given amount of error, predictions are visualized in Figure 16 alongside cutting poses annotated by a human expert.

Model performance improved in the test because additional training data were collected, as well as adjustments were made to DNN architecture. Table 4 presents a comparison of the test set performance for the preliminary model, used during the live test and the final model.

Part of the test also involved making sure that the system ran reasonably well on a laptop that could be brought to the robot cell. The main components involved in running the system were timed, while running simultaneously on a laptop equipped with an Intel i7-8750H CPU. Detection of regions of interest took ≈40 ms, point cloud preparation, including cropping and sampling ≈30 ms and pose prediction ≈40 ms.

### 3.5. Quality of Demonstrations

Because of the difficulty and ambiguity in annotating correct tool poses, it was worth investigating the consistency and correctness of expert demonstrations. To do this, the validation set was annotated twice, and the MAE between the two was calculated. The position component reached an MAE of 0.009 m with a standard deviation of 0.004 m. The cutting plane normals differed by a mean of 5.380° with a standard deviation of 2.587°.

### 3.6. Generalization of the Method

**Figure 17:**
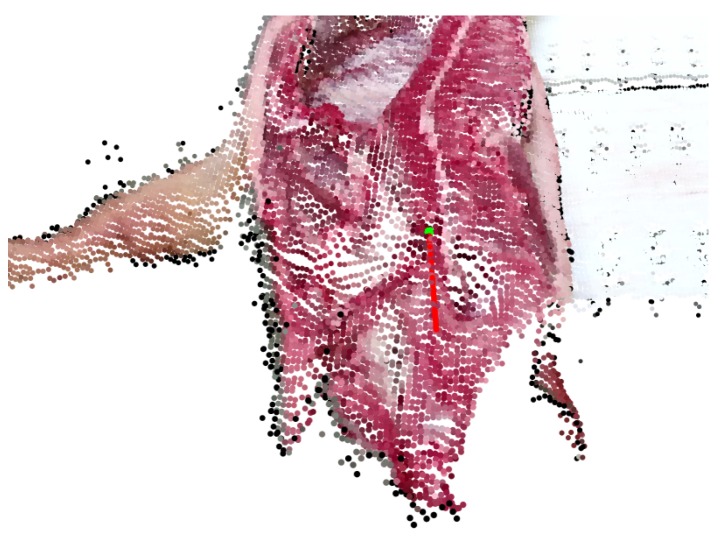
The 5-DoF cutting plane for head cutting illustrated by a green dot and a red normal vector.

The automation of ear cutting is a novel task that has not previously been attempted. A direct comparison with the work of others is therefore not possible. Instead, in this section, we report experiments on how the suggested method generalizes to similar data and how the method performs on very different problems. First, we show preliminary results for predicting the cut that was used to separate the pig’s head from its spine. An example of a predicted cutting pose is shown in Figure 17.

Results are shown in Table 5, which show that the method performed similar to the ear cutting task (see Table 4) despite the small number of training samples. This suggested that the method generalized to similar tasks.

**Figure 18:**
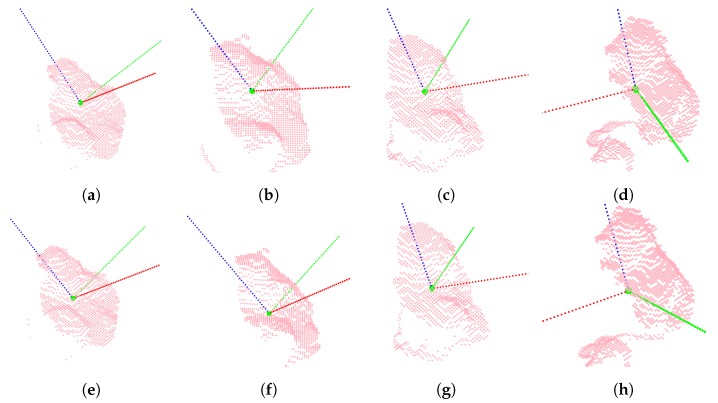
Visualization of object pose. The top row shows ground truth poses, and the bottom row shows predicted poses.

In order to investigate the potential of the method and reveal issues that could guide future improvements, the method was applied on two vastly different public datasets typically used for object pose estimation: LINEMOD [8] and Dex-Net [36]. LINEMOD consists of a total of 15 diverse texture-less 3D objects seen from a wide range of view points. Dex-Net exists in multiple iterations and contains industrial and everyday objects. To perform pose estimation on the LINEMOD dataset, our 5-DoF pose representation was extended to a full 6-DoF representation with two additional normal vectors. We employed the training-test split first suggested by [49] and since employed in [13,39,56]. Fifteen percent of the images were used for training, and the rest were reserved for the test set. A performance comparison is shown in Table 6, for the ape object, and examples of ground truth poses and predictions are shown in Figure 18. Our method was able to predict the position with a mean error of 0.71 cm, but was challenged by the 5° criteria with a mean error of 6.7°. Still without fine-tuning, the method performed similar to the state-of-the-art; see Table 6.

When the pose representation was extended to the full 6-DoF case, the normal vectors began to suffer from the orthonormal constraint of the rotation matrix. This was likely to reduce performance, and alternative representation should be considered for full 6-DoF scenarios. A representation based on Lie algebra promises to solve these issues by representing rotations in a continuous space parameterized by the three scalars in a skew-symmetric matrix [39]. For future applications that require the full 6-DoF, it is worth investigating the value of this and other representations of orientation.

**Figure 19:**
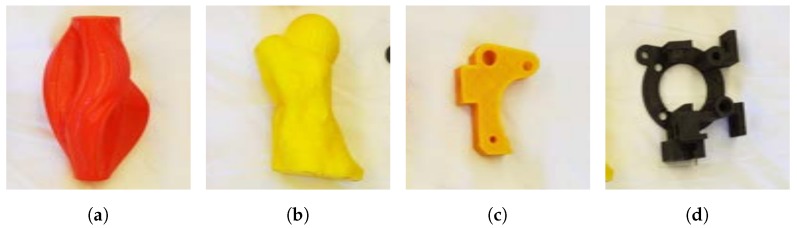
Four out of eight objects in mini-Dex-Net [57].

A fundamentally different problem to the prediction of object or tool pose, where only one correct solution exists, is the scenario where multiple and widely different acceptable solutions exist for the same object. The prototypical task for this kind of scenario is pick-and-place, a task that is found throughout a slaughterhouse. As a representative of this type of task, we used Dex-Net, which is a dataset for grasping rigid objects where our 5-DoF representation was well suited for representing grasps. A grasp could be reduced to either position, when using a suction gripper, or position and 1-DoF rotation for a parallel-jaw gripper [57]. The Dex-Net dataset was distinct in that it was automatically generated through trial and error using a physical robot [36]. The purpose of Dex-Net was not to predict grasps for individual objects, but to learn policies that generalized well to unseen objects. For this reason, Dex-Net, in its entirety, was very large. For the purpose of testing our method, the smaller mini-Dex-Net was used. It consisted of eight objects, four of which are shown in Figure 19.

**Figure 20:**
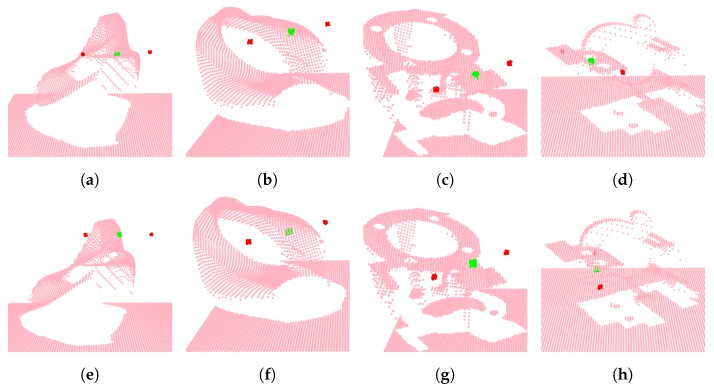
Visualization of grasps. (green) Grasp point; (red) indication of gripper fingers. The top row shows ground truth grasps, and the bottom row shows predictions.

The number of effective grasps ranged between 2085, for the most numerous object, and 114 for the object with the least grasps. A training and test set was created based on the last 25% and first 75% of samples, in each object, respectively. The result was 1592 samples used for training and 4760 for testing. The lack of one true ground truth proved problematic for our method during training due to divergent losses and the difficulty in evaluating whether a prediction was correct when multiple solutions existed. This was reflected by the mean position and angle errors of 1.8 cm and 76.78°, respectively. This type of problem requires a different approach such as the one proposed by the authors of Dex-Net, which classified grasp candidates that were produced based on a range of heuristics [57] or the introduction of an adversarial loss as seen with adversarial imitation learning [58]. Evaluation can only really be done directly on a real robot. A few examples of ground truths and predictions are shown in Figure 20.

## 4. Discussion

The performance measures listed in Table 2 and the distribution of errors across training, validation, and test sets shown in Figure 12 revealed interesting clues about the current limitations and the potential for further improvements. The similarity in the distribution of $P_{0}$ prediction errors across the training and test set suggested that potential gains from increasing the size of the training set were limited. This was supported by the diminishing returns from the increased training set size shown in Figure 15a for $P_{0}$. Further decreases in $P_{0}$ prediction error are likely to be found by improving the accuracy of demonstrations. The distribution of training and test set prediction error for $\vec{nz}$ was less homogeneous. This suggested that, while the DNN was well suited for learning features that described the tool position, this was not the case for the same degree for orientation. At the same time, the continued decrease in the $\vec{nz}$ error in Figure 15b suggested that additional training data might be worthwhile. The significantly poorer performance on the more varied validation set revealed the need for a new or expanded training set in case the setup shown in Figure 5 was changed. There is still much experimentation to be done regarding the architecture of the DNN.

As early as possible, a live test of the system was performed in order to uncover potential issues and to give an indication of the required prediction accuracy. Since 8/11 cuts resulted in acceptable outcomes, it was clear that the system was performing at a level where perhaps very little improvement was needed to reach a satisfactory level of performance. The limited scope of the test made it difficult to conclude exactly how accurate the predictions needed to be, especially considering that the ground truth was not perfect. The sensitivity to random sampling, variations in ROI, and similar small variations were not quantified. However, it was clear that they did have an effect, as predictions jumped around noticeably when predicting tool pose in a live stream of data. The main source of instability was likely the bounding box detector, which was not ideal for precise localization.

The model predictions reached parity with human accuracy levels. This indicated that the quality and consistency of demonstrations became a limiting factor and was a likely reason that our reported accuracy numbers were in line with some results reported for some similar systems [12,51,52] and significantly worse than some others [50]. The differences in applications and the accuracy of the ground truths made a comparison across applications difficult.

### Future Work

The publication of PointNet made all preceding applications of DL to 3D data obsolete. This included many approaches to pose prediction and was supported by significant performance jumps for popular benchmarks such as ModelNet [59] and ShapeNet [60], as well as the clear advantages of the point cloud representation. This paradigm shift was very recent, and only a few applications of PointNet and related methods exist. Much recent work is addressing the limitations of PointNet. For this reason, it would be interesting to incorporate architecture features that add neighborhood processing and hierarchies inspired by CNNs and graph neural networks. Currently, only the positional information from the point clouds is used. However, color information is readily available and may be valuable to the DNN. It at least seems important to the human expert when demonstrating tool poses in VR. In addition to color information, pre-computed point normals may also be of use to the DNN. The most urgent and high payback direction for improvement is expected to be raising the quality of the point cloud data. This includes adjusting the view point to limit the risk of self-occlusion, incorporating multiple view points, and a higher resolution sensor with less noise artifacts. From the results presented in Table 3, a clear performance benefit was found in increased separation between the two types of output. The reason for this was likely that increased weight sharing increased the need for balancing the loss between $P_{0}$ and $\vec{nz}$, which existed in different ranges. Future work should consider introducing and optimizing a scale factor between the two losses, as was done by [55]. It is possible that weight sharing can be beneficial if $P_{0}$ and $\vec{nz}$ are correlated. The presented method was only used to address tasks where a single tool pose is sufficient. However, the method will have to be extended to tasks where multiple tool poses, i.e., a trajectory, are needed. This becomes less straight forward for complex tasks where subsequent tool poses depend on the outcome of those that came before. With the amount of variation that can be expected when operating in a slaughterhouse environment, the system is guarantied to encounter input that differs from the training distribution and will result in faulty predictions. With critical applications, the only solution is to predict when problems are likely to occur and either abstain from acting or ask for human assistance. Alleviating the impact of imperfect models is a major focus of our ongoing work and may involve big data approaches and methods for estimating the confidence in predictions made by DNNs.

## 5. Conclusions

For automation to take over the most demanding and dangerous tasks still performed by humans, there is a critical need for robot controllers that can interpret high-dimensional sensor data and act appropriately under challenging conditions. This work applied the most promising ideas from the fields of pose prediction and recent advances in the application of deep learning methods to 3D data. The ideas and methods were applied to an industrial automation problem that differed from the classical pose prediction tasks that were addressed in most research; namely, by operating on natural objects. For these types of systems to gain widespread use, the training process must be sample efficient, and labeling must be intuitive. For this reason, we attempted to uncover potential bottlenecks and suggest areas with the most potential for improvement. Labeling was made easy and intuitive with the use of virtual reality, only limited by the quality of the collected data. The proposed method predicted tool poses with an average translation error of 0.8 cm and a rotation error of 4.48°. This was achieved with a processing time of approximately 120 ms for a single ear on a laptop CPU. The method’s ability to generalize across problems was shown by its application to other similar tasks including the LINEMOD dataset.

## Figures and Tables

**Table 1:** Dataset statistics.

Name	Frames	Unique Instances	From Unique Viewpoint	Samples
Train set	545	355	724	1632
Validation set	372	116	158	393
Test set	112	75	148	220

**Table 2:** MAE for training, validation, and test sets.

Data Sets	Position Error (std) (m)	Angle Error (std) (°)
Training	0.007 (0.003)	2.025 (1.151)
Validation	0.012 (0.010)	6.555 (4.942)
Test	0.008 (0.004)	4.481 (2.692)

**Table 3:** Validation set mean error for DNN architecture variations.

	Position Error (std) (m)	Angle Error (std) (°)
Full	**0.014 (0.009)**	**6.465 (4.902)**
Early	0.020 (0.009)	6.517 (5.082)
Late	0.029 (0.294)	6.730 (7.504)

**Table 4:** Comparison of the preliminary and final model on the test set.

	Position Error (std) (m)	Angle Error (std) (°)
Preliminary	0.015 (0.008)	4.592 (2.721)
Final	**0.008 (0.004)**	**4.481 (2.692)**

**Table 5:** Prediction error for the principal cut needed for head removal.

Data Sets	Samples	Position Error (std) (m)	Angle Error (std) (°)
Training	188	0.003 (0.002)	0.362 (0.229)
Validation	64	0.013 (0.007)	4.566 (2.460)
Test	64	0.012 (0.006)	3.241 (1.775)

**Table 6:** Performance according to ADD [8] and 5 cm 5° [53] for predicting the pose of ape.

	BB8 (RGB) [56]	Brach (RGB) [49]	Deep-6D (RGB) [39]	DF (RGB-D) [13]	Ours (D)
ADD	40.4	33.2	38.8	79.5	**80.86**
5 cm 5°	**80.2**	34.4	57.8	-	45.90

## Abbreviations

The following abbreviations are used in this manuscript:

Convolutional Neural Network	(CNN)
Deep Learning	(DL)
Deep Neural Networks	(DNN)
Degrees of Freedom	(DoF)
Fully Convolutional Network	(FCN)
Iterative Closest Point	(ICP)
Machine Learning	(ML)
Mean Average Error	(MAE)
Mean Squared Error	(MSE)
Perspective-n-Point	(PnP)
Region of Interest	(ROI)
Virtual Reality	(VR)
